# The Dual Role of Smad7 in the Control of Cancer Growth and Metastasis

**DOI:** 10.3390/ijms141223774

**Published:** 2013-12-05

**Authors:** Carmine Stolfi, Irene Marafini, Veronica De Simone, Francesco Pallone, Giovanni Monteleone

**Affiliations:** Via Montpellier 1, Department of Systems Medicine, University of Tor Vergata, Rome 00133, Italy; E-Mails: irene.marafini@gmail.com (I.M.); ve.desimone@gmail.com (V.D.S.); pallone@uniroma2.it (F.P.)

**Keywords:** TGF-β, colorectal cancer, pancreatic cancer, breast cancer, gastric cancer, epithelial-mesenchimal transition, hepatocellular carcinoma, melanoma, genome-wide association study, carcinogenesis

## Abstract

Smad7 was initially identified as an inhibitor of Transforming growth factor (TGF)-β due mainly to its ability to bind TGF-β receptor type I and prevent TGF-β-associated Smad signaling. More recently, it has been demonstrated that Smad7 can interact with other intracellular proteins and regulate also TGF-β-independent signaling pathways thus making a valid contribution to the neoplastic processes in various organs. In particular, data emerging from experimental studies indicate that Smad7 may differently modulate the course of various tumors depending on the context analyzed. These observations, together with the demonstration that Smad7 expression is deregulated in many cancers, suggest that therapeutic interventions around Smad7 can help interfere with the development/progression of human cancers. In this article we review and discuss the available data supporting the role of Smad7 in the modulation of cancer growth and progression.

## 1. Introduction

Cancer development is a complex process that involves different stages (*i.e*., initiation, promotion, progression). Cancer initiation and promotion are characterized by accumulation of genetic events and multiple host-tumor interactions that ultimately result in uncontrolled cell growth and clonal tumor development [1]. Tumor cell dissemination is a critical feature of cancer progression and involves multiple processes (e.g., decreased cell-cell adhesion, increased motility and invasive properties) that allow cancer cells to detach from the primary tumor, invade the surrounding tissue and generate metastasis at distant sites [2,3].

By regulating a variety of cellular processes such as cell growth, differentiation, apoptosis, migration, cell adhesion, and immune response, the transforming growth factor (TGF)-β signaling pathway controls numerous steps in the development/progression of cancers. In the early stages of tumorigenesis, TGF-β exerts tumor-suppressive action by restricting the growth of epithelial cells and maintaining their differentiation state. On the other hand, in more advanced stages of epithelial tumors, TGF-β acts as a potent driver of cancer progression and metastasis by increasing angiogenesis and inducing epithelial-mesenchymal transition (EMT) [4].

**Figure 1.**
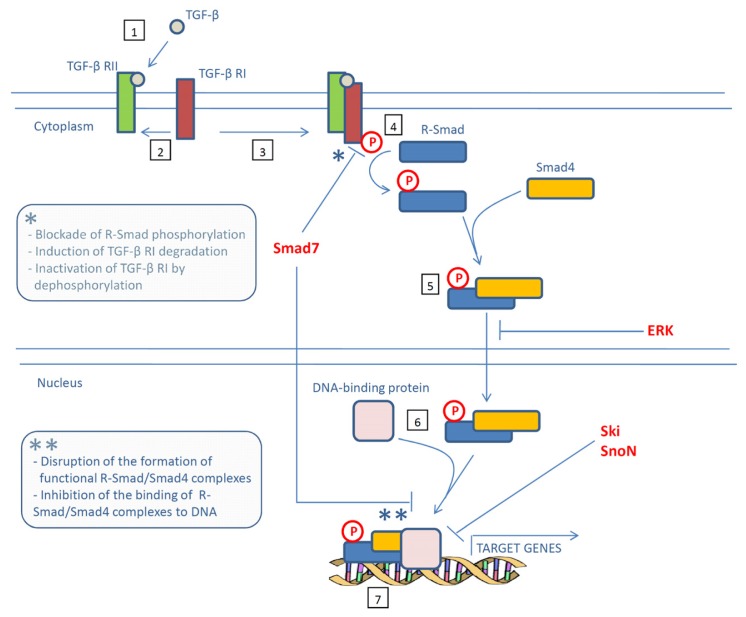
Schematic overview of the transforming growth factor (TGF)-β signaling pathway. Binding of TGF-β to its type II receptor (TGF-β RII) (**1**) attracts the TGF-β type I receptor (TGF-β RI) (**2**) and leads to formation of a receptor complex (**3**) and phosphorylation of TGF-β RI (**4**). Thus activated, TGF-β RI in turn phosphorylates a receptor-activated Smad (R-Smad) (**4**), allowing this protein to associate with Smad4 and move into the nucleus (**5**). Once in the nucleus, this Smad complex associates with DNA-binding proteins (**6**) to activate the transcription of specific target genes (**7**). Negative regulators of this signaling pathway are indicated in red. * and ** indicate mechanisms by which Smad7 antagonizes TGF-β signaling in the cytoplasm and in the nucleus respectively.

TGF-β signaling is initiated by interaction of the cytokine with a complex of heterodimeric transmembrane serine/threonine kinases, consisting of type I (TGF-β RI) and type II (TGF-β RII) receptors, which in turn propagates the signal to a family of intracellular signal mediators known as Smads [5]. Smad proteins are grouped into three functional classes: receptor-activated Smads (R-Smads, including Smad1, Smad2, Smad3, Smad5 and Smad8), common-mediator Smad (*i.e*., Smad4) and inhibitory Smads (*i.e*., Smad6 and Smad7). Once activated through phosphorylation by TGF-β RI, R-Smads form an oligomeric complex with Smad4, which translocates to the nucleus where it modulates the transcription of specific target genes [6]. TGF-β signaling is tightly controlled at multiple levels and negative regulators of this pathway have been implicated in the control of cancer growth and progression (Figure 1).

Extensive phosphorylation of R-Smads by endogenous kinases, including extracellular receptor kinases (ERKs) at specific sites in the region linking the DNA-binding domain and the transcriptional activation domain (*i.e*., linker region), attenuate the nuclear accumulation of these proteins and their ability to mediate TGF-β antiproliferative responses [7]. Ras activation induces the phosphorylation of ERK sites in the linker region of Smad2 and Smad3, and this could explain the loss of growth inhibition by TGF-β in the case of Ras hyperactivation by oncogenic mutations, a common event in several human cancers [7]. Moreover, mutations into the ERK phosphorylation sites of Smad3 resulted in a Ras-resistant form that could rescue the growth inhibitory response of TGF-β in Ras-transformed cells [7].

At the nuclear level, the Smad-binding transcriptional co-repressor Ski and its related protein Ski-related novel gene (SnoN) can interact with Smad2, Smad3 and Smad4 and repress the ability of Smad complexes to regulate expression of target genes [8,9].

While Ski and SnoN are highly expressed in many human cancer cells and tissues where they have been reported to exert pro-oncogenic action, emerging evidence suggests also a tumor suppressor activity for both [10], which could depend on the capacity of these proteins to modulate additional intracellular pathways involved in cancer cell growth.

*Smad7*, also known as *mothers against decapentaplegic homolog 7* (*MADH7*) is located in the chromosome 18 in both human (*i.e*., 18q21.1) and mouse (*i.e.*, 18 51.06 cM) and codifies a protein with 426 aa residues. Smad7 protein structure consists of an *N*-terminal MAD homology 1 (MH1) domain lacking the DNA-binding domain present in Smad4 and most of R-Smads, followed by a non-conserved region called linker and a highly conserved *C*-terminal MAD-Homology 2 (MH2) domain that lacks the SSXS phosphorylation motif present in R-Smads, identified as the target of receptor-dependent phosphorylation (please see refs. [11,12] for more detailed information).

Smad7 antagonizes TGF-β signaling through multiple mechanisms both in the cytoplasm and in the nucleus. For example, Smad7 blocks R-Smad phosphorylation by occupying the catalytic domain of TGF-β RI [13,14]. Smad7 also induces degradation of TGF-β RI through recruitment of Smurf1/2 or Nedd4-2, some of the E3 ubiquitin ligases that target activated TGF-β receptor complexes for degradation via proteasome [15–17]. Moreover, Smad7 interacts with growth arrest and DNA damage protein (GADD34), a regulatory subunit of the protein phosphatase 1 (PP1) holoenzyme, thereby leading to TGF-β RI inactivation by dephosphorylation [18]. At nuclear level, Smad7 can exert its inhibitory activity by disrupting the formation of functional R-Smad/Smad4 complexes as well as their binding to DNA [19].

In addition to its role in the negative regulation of TGF-β signaling, Smad7 modulates other intracellular pathways in both TGF-β-dependent and -independent manner [11]. For instance, Smad7 promotes tumor necrosis factor (TNF)-induced apoptosis through the inhibition of expression of antiapoptotic NF-κB target genes [20]. Smad7 plays an important role in TGF-β-induced negative regulation of Interleukin-1/Toll-like receptor (IL-1R/TLR) signaling through binding to Pellino-1, an adaptor protein of interleukin-1 receptor associated kinase 1 (IRAK1). Smad7-Pellino-1 interaction blocks the formation of the IRAK1-mediated IL-1R/TLR signaling complex thus abrogating NF-κB activity and reducing the expression of pro-inflammatory genes [21]. Moreover, Smad7 affects NF-κB activity by regulating either directly or indirectly TGF-β-activated kinase 1 (TAK1) activation [22,23]. Smad7 can antagonize Wnt signaling by forming complexes with β-catenin and Smurf2 thereby promoting β-catenin degradation via proteasome [24]. The association of Smad7 with β-catenin also plays a pivotal role in the regulation of some transcription factor involved in tumorigenesis (e.g., c-myc) [25].

Due to these abilities, Smad7 affects numerous processes which are important for cell homeostasis and observations derived from Smad7 transgenic mice suggest that Smad7 is involved in the modulation of immune responses [26], embryo cardiac development and cardiac function [27], early development and organogenesis [28], and skeletal muscle cell differentiation [29,30].

**Figure 2.**
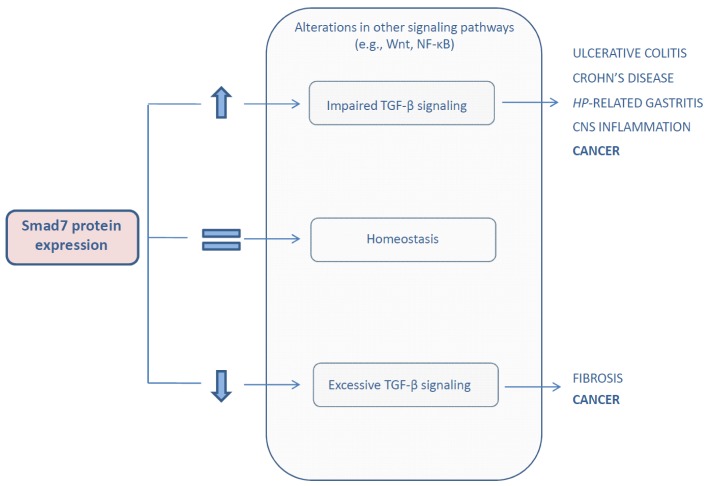
Schematic overview depicting the pathogenic roles of a deregulated Smad7 protein expression. Abbreviations: CNS, central nervous system; *HP*, *Helicobacter-pylori*.

Interestingly, a deregulated Smad7 protein expression has been documented and supposed to play a pathogenic role in a variety of human disorders (Figure 2).

For example, Smad7 expression is down-regulated in fibrotic tissues and implicated in the progression of fibrosis in different organs such as pancreas, liver, lung, skin, and kidney [11] in line with the profibrotic actions of the TGF-β pathway.

On the other hand, Smad7 is over-expressed and limits TGF-β-mediated anti-inflammatory signals during inflammation of the central nervous system [31], in inflammatory bowel diseases (IBD), such as ulcerative colitis and Crohn’s disease, as well as in *Helicobacter-pylori*-related gastritis [32,33].

Increasing evidence indicates that Smad7 is differently expressed in human cancers [34–36], and it could either sustain or restrain cancer cell growth [11]. Here, we review the data supporting the dual role of Smad7 in the control of carcinogenesis.

## 2. Expression and Role of Smad7 in Cancer

Smad7 can be transcriptionally regulated by TGF-β, epidermal growth factor (EGF) and inflammatory cytokines, such as TNF-α, IL-1β and IFN-γ in different cell lines [37–40]. In contrast, little is known about its regulation in human tissues. Studies in patients with IBD have shown that Smad7 can be also regulated at post-transcriptional level by mechanisms that enhance acetylation on lysine residues thus reducing ubiquitination-mediated proteasomal degradation [41]. High Smad7 is seen in several human malignancies and there is preliminary evidence that Smad7 expression correlates with the clinical prognosis of cancer patients. For instance, Smad7 up-regulation was associated with poor survival rate in esophageal squamous cell carcinoma and a shorter time to recurrence in endometrial carcinoma [42,43]. However, a different scenario has emerged when Smad7 has been investigated in other tumors. In particular, it was shown that ectopic expression or deletion of Smad7 in cancer cells can differently regulate tumorigenesis depending on the cell context analyzed.

The pro- and anti-tumorigenic effects of Smad7 in different cancer types are summarized in the Table 1 and discussed below.

### 2.1. Colorectal Cancer

*Smad7* gene variants have been extensively analyzed in patients with colorectal cancer (CRC). Boulay *et al*., found that CRC patients with deletion of *Smad7* had a favorable clinical outcome compared with patients with *Smad7* amplification [44]. More recently, further genetic variants within *Smad7* gene have been linked to colorectal carcinogenesis in two genome-wide association studies (GWAS) [65,66]. In both studies, a highly significant association with CRC was found for two single nucleotide polymorphisms (SNPs) in *Smad7* (*i.e*., rs4939827, rs12953717). The association of these SNPs with CRC was then confirmed by two other GWAS [67,68].

To clarify the role of Smad7 in CRC cell growth, Halder *et al*., overexpressed Smad7 in FET, a CRC cell line, and showed that Smad7 enhanced anchorage-independent cell growth, favored the formation of colonies on soft agar and increased resistance against apoptosis through a mechanism dependent on suppression of TGF-β signaling. Smad7-overexpressing FET cells showed an increased tumorigenicity when injected subcutaneously into immunodeficient nude mice [45] as compared to control FET cells. The same group assessed the role of Smad7 in colon cancer metastasis and demonstrated that injection of Smad7-overexpressing FET cells in the spleen of athymic nude mice favored the development of liver metastasis [46]. The pro-metastatic role of Smad7 was associated with increased expression of junctional proteins (e.g., E-cadherin, Claudin-1, Claudin-4) at distant sites. These findings are in line with our recent studies showing that Smad7 is over-expressed in human CRC and inhibition of Smad7 with a specific Smad7 antisense oligonucleotide reduces CRC cell growth both *in vitro* and *in vivo* in mice (personal unpublished observations). The factors/mechanisms underlying Smad7 upregulation in CRC cells are not yet known. It has been recently reported that microRNA-25 (miR-25), whose expression is down-regulated in CRC tissue, is a negative regulator of Smad7, raising the possibility that in CRC cells high Smad7 can be linked to the low content of miR-25 [69].

As pointed out above Smad7 is also over-expressed by immune cells in IBD tissue and studies in experimental models have shown that over-expression of Smad7 in T cells associates with severe colitis and reduced growth of colitis-associated CRC, thus highlighting the opposing role of Smad7 in the control of sporadic and colitis-associated CRC [47].

### 2.2. Pancreatic Cancer

An early study by Kleeff and colleagues showed that Smad7 RNA transcripts were increased in human pancreatic cancer as compared to the normal pancreas [34]. To determine the role of Smad7 in pancreatic cancer cells, COLO-357 cells were stably transfected with a full-length Smad7 expression vector. Smad7 overexpressing COLO-357 cells were resistant to the TGF-β-mediated growth inhibition *in vitro* and exhibited a marked capacity to form colonies in soft agar and tumors in nude mice [34]. Studies investigating the mechanisms underlying the pro-tumorigenic effects of Smad7 identified thioredoxin-1 and retinoblastoma as key molecules involved in the Smad7-dependent aggressiveness of pancreatic cancer cells [70,71].

More recently, Kuang and co-workers provided *in vivo* evidence that Smad7 is implicated in the early stages of pancreatic cancer. Using a transgenic mouse with pancreas specific Smad7 overexpression, these authors reported that Smad7 blocked TGF-β signaling in the pancreas and induced premalignant ductal lesions with the characteristics of pancreatic intraepithelial neoplasia (PanIN), the precursor stage to pancreatic carcinoma [48]. However, Wang and co-workers showed that high expression of Smad7 in pancreatic cancer associated with a more favorable prognosis compared with patients with lower levels of Smad7 who exhibited increased incidence of lymph node metastasis and liver metastasis after surgery [49]. The reason for this apparent difference is not yet known even though it is conceivable that such a discrepancy could be at least in part due to the ability of Smad7 to interfere with the opposing role of TGF-β in pancreatic tumor initiation and progression.

### 2.3. Gastric Cancer

The expression of Smad7 in gastric cancer progression and its prognostic significance was initially investigated by Kim and colleagues. By immunohistochemistry, it was demonstrated that 98 out of 304 patients (32.2%) who had undergone gastrectomy expressed Smad7 in gastric cancer tissues whereas no expression was detected in normal tissues [51]. This later result is however surprising as we had detected Smad7 in normal gastric mucosa by Western blotting and real-time PCR [33] and Leng and co-workers documented a constitutive expression of Smad7 in the normal gastric mucosa [50]. This group showed also up-regulation of Smad7 in gastric cancer and peri-tumoral area, particularly in poorly differentiated tumors and in those with lymphatic metastasis [50]. It has been also reported that gastric cancer patients with elevated levels of Smad7 had a poor prognosis independently of other well-established clinical prognostic factors, such as tumor size, depth of invasion and lymph node metastasis [51]. Consistent with this is the demonstration that ectopic Smad7 expression increases the survival of SGC7901 gastric cancer cells [50].

Altogether, these findings highlight the involvement of Smad7 in gastric tumorigenesis.

### 2.4. Skin Cancer

Human papilloma and squamous cell carcinoma (SCC) express elevated levels of Smad7 as compared to normal epidermis [36]. Using a mouse model of chemically-induced skin carcinogenesis, Liu *et al*., reported that Smad7 overexpression in H-ras-transduced keratinocytes determined the conversion of benign to malignant epithelial cells and a rapid progression to squamous cell carcinoma [52]. This effect was associated with a marked increase in cell proliferation, inhibition of TGF-β signaling and induction of EGF family members, which regulate various signals associated with tumor growth and metastasis [72]. Moreover, using a xenograft model in which primary keratinocytes mixed with dermal fibroblasts are grafted into nude mice, the same authors reported that H-ras/Smad7, but not H-ras, keratinocytes progressed to SCC [52].

In contrast, two studies by Mauviel’s group reported a TGF-β-dependent tumor-suppressive role of Smad7 in metastatic melanoma cells [53,54]. Stable over-expression of Smad7 in 1205Lu cells reduced production of tissue-degrading proteases and hence the invasive capacity and the *in vitro* anchorage-independent growth as well as tumor formation following subcutaneous injection in nude mice [53]. In a model of bone metastases induced by inoculation of tumor cells into the left cardiac ventricle of nude mice, Javelaud *et al*., showed that animals injected with Smad7-transfected 1205Lu cells had significantly less osteolytic metastases and longer survival compared with mice injected with parental and mock-transfected 1205Lu cells [54]. These changes were accompanied by a reduced secretion of gelatinases and diminished expression of metastasis-related molecules (e.g., interleukin-11, CXCR4, osteopontin). DiVito and co-workers, using an *in vivo* human skin grafting system, showed that Smad7-expressing 1205Lu cells positioned themselves proximal to the dermal-epidermal junction and failed to form tumors, while control cells invaded the dermis and formed tumors [55]. Mechanistically, it was proposed that Smad7 promoted heterotypic cell-cell interactions through the redistribution of cell adhesion proteins to the cell surface thereby mitigating tumor invasion [55]. Altogether these data suggest that Smad7 can be both pro- and anti-tumorigenic in the skin.

### 2.5. Breast Cancer

Theohari and colleagues investigated the expression of Smad7 in 150 invasive breast carcinoma specimens and showed that Smad7 levels positively correlate with tumor size, stage, matrix metalloproteinase (MMP)-9 and MMP-14 expression thus resulting in an aggressive phenotype [56]. However, experimental evidence suggests that Smad7 can regulate either positively or negatively breast carcinogenesis. In a model of breast cancer metastasis, Azuma *et al*., reported a decrease in lung and liver metastasis and longer survival when mice were intravenously injected with Smad7-transfected mouse mammary carcinoma JygMC(A) cells compared to mice injected with mock-transfected JygMC(A) cells. These effects were suggested to rely on the increased expression of major components of adherent and tight junctions (e.g., E-cadherin) and decreased expression of promigratory cadherins (e.g., *N*-cadherin) in JygMC(A) cells that expressed exogenous Smad7 [57]. Hong and colleagues showed that over-expression of Smad7 sensitized MCF7 breast cancer cells to TNF-induced cell death and associated this effect with inhibition of expression of antiapoptotic NF-κB target genes [20]. Finally, Smad7 was suggested to negatively regulate the EGF signaling pathway in breast cancer cells as ectopic Smad7 expression in SKBR3 cells completely abrogated EGF-induced MMP-9 expression [40].

In contrast with the above findings, two recent studies suggested that Smad7 is an inhibitor of EMT and cell invasion in breast carcinogenesis. Papageorgis and co-workers delineated a link between Smad7 and the maintenance of epigenetic silencing of epithelial genes during EMT of breast cancer cells. Using breast cancer cell lines derived from a common genetic background (*i.e*., MCF10A) which accumulated distinct genetic/epigenetic alterations *in vivo* thus acquiring properties associated with gradual progression from nontumorigenic to carcinogenic state, these authors showed that Smad7 overexpression suppresses migration and invasion of mesenchymal-like MCF10CA1h cells, a malignant variant of Ras-transformed MCF10A cells, by reversing the DNA methylation status of specific epithelial markers (*i.e*., E-cadherin, γ-catenin, and β-catenin) thus inducing their re-expression [58].

Along the same line is the work by Smith *et al.*, who showed that the miR-106b-25 cluster negatively regulates Smad7 expression thereby activating TGF-β signaling and inducing EMT in MCF7 cells [59].

### 2.6. Liver Cancer

Park *et al*., evaluated Smad7 in the different phases of hepatocellular carcinoma (HCC) development and documented Smad7-expressing tumor cells in 25 out of 41 (61%) advanced tumors whereas no Smad7-positive cells were detected in dysplastic nodules and early HCCs [73]. Using a mouse model of HCC induced by diethylnitrosamine (DEN), Wang and colleagues showed that Smad7-deficient mice had higher tumor incidence and multiplicity than wild-type mice. Moreover, tumor cells from Smad7 KO mice displayed increased proliferation, diminished apoptosis and higher colony formation compared with those from wild-type littermates. Deletion of Smad7 increased cell growth of primary HCC cells while ectopic expression of Smad7 in HCC cell lines markedly suppressed cell growth and colony formation. These effects were associated with the Smad7-mediated inhibition of the G1-S phase transition and induction of apoptosis through attenuation of NF-κB and TGF-β signaling [60].

Xia *et al*., confirmed the decreased expression of Smad7 in HCC samples, particularly in patients with early recurrence and poor prognosis [61]. The same group also showed that, in HCC, Smad7 is inversely related to miR-216a/217 cluster, a negative regulator of Smad7, which controls EMT and cell migration of HCC cells [61]. Altogether, these observations suggest that Smad7 may act as a tumor suppressor in HCC.

### 2.7. Prostate Cancer

Evidence linking Smad7 and prostate cancer comes from the work of Landstrom’s group. Initial studies showed that Smad7 was expressed in rat prostate cancer cells undergoing apoptosis [74] and ectopic Smad7 expression induced apoptosis of PC-3U human prostate cancer cells [62]. Further investigation revealed that Smad7 acted as a scaffold protein to facilitate TGF-β-induced activation of p38 and subsequent apoptosis [75] and was required for induction of apoptosis by the anti-cancer agent 2-Methoxyestradiol [63]. More recently, Ekman *et al*., showed that Smad7 forms a complex with APC in a p38-dependent fashion and facilitates TGF-β-induced accumulation of β-catenin, thereby promoting migratory responses in prostate cancer cells [64].

## 3. Conclusions

The data described in this article indicate that Smad7 can have both pro- and anti-tumor actions depending on the cancer type analyzed.

Given the role of Smad7 in inhibiting TGF-β signaling, a possible explanation of these opposite effects could be due to the different functionality of this pathway among distinct cancer types. Indeed, while mutations in TGF-β signaling machinery are rare in most cancers, frequent genetic alterations in Smad components characterize gastrointestinal carcinomas (e.g., pancreatic, colorectal) [76–78] and suggest a tumor suppressive and anti-metastatic role of TGF-β pathway in a context dependent manner [79–82].

Moreover, even in the same tumor the function of Smad7 can switch from tumor-suppressive to tumor-promoting depending on the tumor stage (*i.e*., early versus advanced). These apparently contradictory functions are not surprising, given the opposite role of TGF-β signaling pathway in early versus advanced tumor stages and the interaction of Smad7 with a vast array of functionally heterogeneous molecules that may be differently expressed during the carcinogenetic process.

Further work is needed to delineate the mechanisms by which Smad7 exerts its distinct functions on tumorigenesis and to clarify which cancer-related pathways are predominantly affected by Smad7 in the different contexts. It would be also useful to study the role of Smad7 in experimental models of metastasis deriving from primary tumors that evolve *in situ* and more closely resemble the human disease as well as the effect of Smad7 inhibition in cancer cells that constitutively express this protein. This information will ultimately help us to understand the complex role of Smad7 in the different phases of carcinogenesis and eventually pave the way for the development of therapeutic strategies which, through modulation of Smad7 function, can contribute to attenuate/halt the course of these diseases.

## Figures and Tables

**Table 1.** Pro- and anti-tumor effects of Smad7.

Cancer type	Method	Observation	Ref.
Endometrial	Observational study	Smad7 upregulation associates with poor survival rate	[42]
Esophageal	Observational study	Smad7 upregulation associates with shorter time to recurrence	[43]
Colorectal	Observational study	CRC patients with deletion of Smad7 have a favorable clinical outcome compared with patients with Smad7 amplification	[44]
Colorectal	Colony formation assay, xenografts induced by FET cells in immunodeficient mice	Smad7-overexpressing FET cells show aggressive colony formation on soft agar and increased tumorigenicity *in vivo* compared with control FET cells	[45]
Colorectal	Metastasis induced by the injection of FET cells in the spleen of immunodeficient mice	Injection of Smad7-overexpressing FET cells results in the development of liver metastasis	[46]
Colorectal	AOM + DSS-driven colitis associated CRC	Over-expression of Smad7 in T cells associates with severe colitis and reduces the growth of colitis-associated CRC	[47]
Pancreatic	Colony formation assay, xenografts induced by FET cells in immunodeficient mice	Smad7 overexpressing COLO-357 cells are resistant to the TGF-β-driven growth inhibition *in vitro* and exhibit a marked increase in their capacity to form colonies in soft agar and tumors in nude mice	[34]
Pancreatic	Transgenic mouse with pancreatic overexpression of Smad7	Smad7 blocks TGF-β signaling in the pancreas and induces premalignant ductal lesions with the characteristics of pancreatic intraepithelial neoplasia	[48]
Pancreatic	Observational study	Expression of Smad7 associates with a more favorable prognosis compared with patients with lower levels of Smad7 who exhibited increased incidence of lymph node metastasis and liver metastasis after surgery	[49]
Gastric	Observational study	Elevated Smad7 levels in tumors with lymphatic metastasis	[50]
Gastric	Observational study	Patients bearing tumors with positive Smad7 expression have a poor prognosis	[51]
Gastric	Cell culture	Ectopic Smad7 expression increased the survival of SGC7901 gastric cancer cells	[50]
Skin	Mouse model of chemically-induced skin carcinogenesis	Smad7 overexpression in H-ras-transduced keratinocytes determines the conversion of benign to malignant epithelial cells and a rapid progression to squamous cell carcinoma	[52]
Skin	Xenograft model in which primary keratinocytes mixed with dermal fibroblasts are grafted into nude mice	H-ras/Smad7 but not H-ras keratinocytes progresses to SCC	[52]
Skin	Colony formation assay, xenografts induced by 1205Lu cells into immunodeficient mice	Stable over-expression of Smad7 in 1205Lu cells reduces MMP-2 and MMP-9 production, invasive capacity and anchorage-independent growth *in vitro* as well as subcutaneous tumor formation in nude mice	[53]
Skin	Model of bone metastases in which tumor cells are inoculated into the left cardiac ventricle of nude mice	Animals injected with Smad7-transfected 1205Lu cells have significantly less osteolytic metastases and longer survival compared with mice injected with parental and mock-transfected 1205Lu cells	[54]
Skin	*In vivo* human skin grafting system	Smad7-expressing 1205Lu cells position proximal to the dermal-epidermal junction and fail to form tumors, while control cells form tumors within the dermis	[55]
Breast	Observational study	Smad7 expression correlates with a poor prognosis in patients with invasive breast carcinoma	[56]
Breast	Breast cancer metastasis induced by intravenous injection of mouse mammary carcinoma JygMC(A) cells	Mice injected with Smad7-transfected JygMC(A) cells show fewer lung and liver metastasis and longer survival than mice injected with mock-transfected JygMC(A) cells	[57]
Breast	Cell culture	Smad7 sensitizes MCF7 breast cancer cells to TNF-induced cell death	[20]
Breast	Cell culture	Ectopic Smad7 expression in SKBR3 cells completely abrogates EGF-induced MMP-9 expression	[40]
Breast	Cell culture	Smad7 overexpression suppresses migration and invasion of mesenchymal-like MCF10CA1h cells	[58]
Breast	Cell culture	miR-106b-25 cluster negatively regulates Smad7 expression thereby activating TGF-β signaling and inducing EMT in MCF7 cells	[59]
Liver	Mouse model of HCC induced by DEN	Smad7-deficient mice have higher tumor incidence and multiplicity than wild-type mice	[60]
Liver	Observational study	Low Smad7 expression in HCC samples associates with better disease free survival	[61]
Liver	Cell culture	Smad7 restrains EMT and cell migration of HCC cells	[61]
Prostate	Cell culture	Ectopic Smad7 expression induces apoptosis in PC-3U human prostate cancer cells	[62]
Prostate	Cell culture	Smad7 is required for the induction of apoptosis by the anti-cancer agent 2-Methoxyestradiol in PC-3U cells	[63]
Prostate	Cell culture	Smad7 promotes migratory responses in PC-3U cells	[64]

White background = pro-tumorigenic effect; grey background = anti-tumorigenic effect; Abbreviations: AOM, azoxymethane; CRC, colorectal cancer; DEN, diethylnitrosamine; DSS, dextran sodium sulfate; EGF, epidermal growth factor; EMT, epithelial-mesenchymal transition; HCC, hepatocellular carcinoma; MMP, metalloproteinase; TGF, transforming growth factor; TNF, tumor necrosis factor.
